# Five-Year Analysis of Surgical Site Infections in Three Orthopaedics and Trauma Wards under HAI-Net from the South of Poland in 2014–2018 Considering the Standardized Infection Ratio

**DOI:** 10.3390/medicina58050682

**Published:** 2022-05-20

**Authors:** Marta Zofia Wałaszek, Roża Słowik, Adam Domański, Michał Jan Wałaszek, Anna Różańska, Małgorzata Kołpa

**Affiliations:** 1 State Higher Vocational School in Tarnów, 33-100 Tarnów, Poland; mz.walaszek@gmail.com (M.Z.W.); rozaslowik@interia.eu (R.S.); malgorzatakolpa@interia.pl (M.K.); 2 St. Luke’s Provincial Hospital in Tarnów, 33-100 Tarnów, Poland; 3 Department of Distributed Systems and IT Equipment, Faculty of Automatic Control, Electronics and Computer Science, The Silesian Technical University, 44-100 Gliwice, Poland; adam.domanski@polsl.pl; 4 Polish Society of Hospital Infections, 31-121 Kraków, Poland; mj.walaszek@gmail.com; 5 Department of Microbiology, Jagiellonian University, 31-121 Kraków, Poland

**Keywords:** surgical site infections, hip prosthesis, knee prosthesis, open reduction of fracture, close reduction of fracture, infection control

## Abstract

*Background and Objectives*: Surgical site infections (SSIs) are the most common healthcare-associated infections (HAIs) in surgical wards. The highest risk of developing SSI is carried by operations involving implants, such as: hip prosthesis (HPRO), knee prosthesis (KPRO), open reduction of fracture (FX), and closed reduction of fracture with internal fixation (CR). Objectives. The objective of the study was to assess the incidence of SSI in patients subjected to HPRO, KPRO, FX, and CR procedures in orthopaedics and trauma wards in 2014–2018 considering risk factors included in the SIR index. *Materials and Methods*: The study included 6261 patients who were subjected to orthopaedic surgery in 2014–2018. The investigation covered three hospitals with orthopaedics and trauma wards. The research was conducted in the framework of the national HAI surveillance programme according to the methodology of the HAI-Net, ECDC. *Results*: A total of 6261 surgeries were investigated, of which 111 cases of SSI were detected. The incidence was 1.8%; HPRO (incidence 2.1%, median (Me) surgery duration 90 min, and standardized infection ratio (SIR) above 1 in all units tested); KPRO (incidence 2.0%, Me 103 min, and SIR above 1 for all units tested); FX (incidence 1.9%, Me 70 min, and SIR above 1 for two units tested and below 1 in one unit); CR (incidence 1.0%, Me 55 min, and SIR—not calculated). The etiological agents that were most frequently isolated from patients with SSI were Staphylococcus aureus, coagulase-negative Staphylococcus, and Klebsiella pneumoniae. *Conclusions*: HPRO, KPRO, and FX operations performed in the studied wards carried a higher risk of developing SSI than that predicted by SIR. SSIs accounted for a significant percentage of the overall infection pool in CR surgeries. Actions should be undertaken to reduce the incidence of SSI in these surgeries. There should be a hospital network which facilitates cooperation in order to better monitor and analyse the incidence of SSI.

## 1. Background

Surgical site infections (SSIs) represent the most common infections in surgical wards, and they are infections of the surgical site which may occur in three different types. European studies have found that SSIs account for 27% of all healthcare-associated infections (HAIs) [1]. Similarly, in a 5-year (2012–2016) single-centre study from Poland, SSIs comprised 23% of all HAIs detected [2]. A similar incidence was observed in Spain, where SSIs constituted 27% of the total HAI pool [3]. Incidence rates of SSI vary depending on the degree of surgical site microbiological classification and type of orthopaedic surgery and range from 2% in clean surgical sites (e.g., HPRO, KPRO), 5.7% in clean-contaminated sites (e.g., blunt trauma), 10.9% in contaminated sites (e.g., open traumatic wound), to 6.9% in dirty sites (e.g., procedures performed on necrotic tissue) [1]. Incidence of SSI in orthopaedics and trauma wards differs between countries or even in specific regions; in Poland, it ranges from 2.7% to 6.6% [4,5]. The global literature estimates the total incidence of SSI in orthopaedic patients in the range from 0.9% to 22.7% [6,7]. However, interpretation of the incidence rates should include the analysis of the specific types of SSI. There can be three different types of SSI: superficial, deep, and organ-space [8]. They are the same for all types of surgery.

Orthopaedic wards specifically perform operations with varying degrees of cleanliness of the surgical site, with a clear predominance of clean sites. Surgeries involving bone tissue, which is extremely sensitive to infection, are more complex and require compliance with the rules of the sanitary regime and the use of complicated surgical instruments. The use of biomaterial implants in orthopaedics significantly improves the quality of life of patients; however, it poses a serious risk of developing SSIs. Despite the application of preventive measures against infections and improvement of surgical techniques, SSIs are still the primary cause of failures in orthopaedics [9]. Patients with SSI have worse treatment outcomes and prolonged treatment duration. Their health condition may deteriorate which, as a consequence, can lead to returning to professional work later, disability, or even death. Therefore, epidemiological analyses of SSIs in this type of surgery are necessary. However, they require the calculation of not only simple incidence rates but also rates stratified by risk factors. This type of approach makes it possible to understand the epidemiological situation in detail, as well as benchmarking with the availability of data from various centres. The most common approach for SSIs is calculation of the standardized infection ratio (SIR) which is a summary measure used to track infections at a national, state, or local level over time. SIR compares the actual number of infections reported to the number that is predicted, given the standard population, adjusting for several risk factors that have been found to be significantly associated with differences in infection incidence. The standardized infection ratios (SIRs) for the development of SSI according to the National Healthcare Safety Network (NHSN) were collected, based on three most important variables affecting the probability of SSI, and these concerned: (a) degree of surgical site cleanliness, (b) duration of surgery, and (c) patient condition according to the ASA score, which is one of the widely used methods for such detailed analyses that was previously used by authors in single-centre study focusing only on hip and knee arthroplasties [10].

An earlier single-centre study conducted by the authors in 2012–2018 with the use of the standardized infection ratio (SIR) found a higher than expected incidence of SSI in the ward under study [9]. The results of this study were an inspiration to extend the scope of research to include additional orthopaedics and trauma wards in the region.

OBJECTIVES: The objective of the study was to assess the incidence of SSI in patients subjected to hip prosthesis (HPRO), knee prosthesis (KPRO), open reduction of fraction (FX), and close reduction of fracture (CR) procedures in orthopaedics and trauma wards in 2014–2018 considering risk factors included in the SIR index.

## 2. Material and Methods

We performed a retrospective multicentre study including patients undergoing surgery in three orthopaedics wards in Poland. All consecutive patients aged over 18 years undergoing procedures listed above were included in the analysis. There were no specific criteria for exclusion, because surveillance was realised as a part of routine work of study wards and hospitals.

Hip prosthesis, knee prosthesis, and open and close reduction of fractures were covered by the surveillance, registration, and analysis. The International Classification of Diseases, Ninth Revision, Clinical Modification (ICD-9-CM), for these procedures are as follows: hip prosthesis—HPRO (ICD-9: 00.70–00.73; 00.85–00.87; and 81.51–81.53), knee prosthesis—KPRO (ICD-9: 00.80–00.84; 81.54; and 81.55), and other musculoskeletal surgeries: open reduction of fracture—FX (ICD-9: 79.21; 79.22; 79.25; 79.26; 79.31; 79.32; 79.35; 79.36; 79.51; 79.52; 79.55; and 79.56), and closed reduction of fracture—CR (CDI-9: 79.11–79.18; 79.191–79.194).

The studied hospitals participated in the nationwide voluntary system of active registration of hospital infections run by the Polish Society of Hospital Infections, consistent with the methodology of the Healthcare-Associated Infections Surveillance Network (HAI-Net), European Centre for Disease Prevention and Control (ECDC) [8]. In 2014–2018, these hospitals detected, classified, and registered infections according to the ECDC definitions [8,11], which were created on the basis of the methodology of the National Healthcare Safety Network (NHSN) [12]. Infections qualified as SSI developed within 1 year of surgery in case of deep or organ-space infection together with implantation or within 30 days in other cases, according to the definitions in effect at that time in the countries of the European Union (EU) [13]. These were 3 hospitals located in the south of Poland: Hospital no. 1 with the trauma and orthopaedic surgery ward (H1); Hospital no. 2; with the orthopaedics and traumatology of the locomotor system ward (H2); and Hospital no. 3 (H3) with the orthopaedics and trauma ward (H3).

Hospital 1 (H1) has participated in the programme of active surveillance of infections since 2012. The orthopaedics and trauma ward is focused on the treatment of locomotor injuries and especially long bone fractures using internationally recognised methods. The leading procedures are implantations of hip and knee joint prostheses as well as repair operations using allogeneic and own bone grafts. The unit has two- and three-person rooms with 40 orthopaedic beds. The ward provides constant, 24 h, care pertaining to motor organ trauma surgery for the city of Tarnów and its surrounding areas.

Hospital 2 (H2) has participated in the programme of active surveillance of infections since 2012. The orthopaedics and traumatology of the locomotor system ward offers a wide range of surgical procedures, including arthroscopic operations, primary endoprosthesis, and revision hip joint arthroplasty. It has novel surgical methods at its disposal. The department has two- and three-person patient rooms with 42 orthopaedic beds. The ward provides constant, 24 h, care pertaining to motor organ trauma surgery for the town of Gorlice and its surrounding areas.

Hospital 3 (H3) has participated in the programme of active surveillance of infections since 2012. The orthopaedics and trauma ward offers highly specialised services concerning orthopaedics and traumatology, including hip arthroplasty, knee arthroplasty, surgical fracture setting, and fracture stabilisation. The unit has two- and three-person rooms with 40 orthopaedic beds. The ward provides constant, 24 h, care pertaining to motor organ trauma surgery for the city of Bielsko-Biała and its surrounding areas.

Data were collected using two paper forms from the Polish Society of Hospital Infections programme: the operation form and the infection form, filled in the wards or in the operating rooms. After, data were transferred into an electronic database, with unique access for each setting.

Basic demographic data on patients and their stay in the wards were collected: age, sex, time of admission, and time of operation. However, according to protocol, additional data necessary for calculation of the standardized infection ratio (SIR) for the development of SSI according to the National Healthcare Safety Network (NHSN) were also collected. There were 3 variables affecting the probability of SSI and these concerned: (a) degree of surgical site cleanliness, (b) duration of surgery, and (c) patient condition according to the ASA score. The degree of surgical site cleanliness was determined by the operating surgeon in the course of the procedure, and therefore, 1 point was assigned for a contaminated (W3) or dirty (W4) surgical site. Then, 1 point was given to a patient whose surgery duration exceeded the 75th percentile. Assessment according to the ASA score was conducted by an anaesthesiologist prior to the operation and 1 point was assigned when the ASA score was 3 or higher. The SIR could, therefore, range from 0 to 3, and when SIR reaches more than 1, it means that there were more SSIs than predicted.

Statistical analysis was conducted using basic statistical parameters, i.e., mean, median, standard deviation (SD), and 95% confidence intervals (95%CI). The level of significance was *p* < 0.05.

Statistical analysis of the material collected made use of the IBM SPSS statistical software (SPSS—Statistical Package for the Social Sciences) STATISTICS 24, Armonk, NY, USA and Microsoft Excel Microsoft Office 2016 Redmond, WA, USA.

The study followed the Declaration of Helsinki guidelines (2008). The use of data was approved by the Bioethics Committee of the Jagiellonian University (No. KBET /122.6120.118.2016 from 25.05.2016). All the data entered into the electronic database and analysed in this study were previously anonymised.

## 3. Results

The total number of patients covered by the study was 6261, and 3540 (56.5%) were women. There were 111 cases of SSI detected. The wards under study most often performed HPRO procedures (35%), followed by FX (34%), CR (22%), and KPRO (8.9%). The overall incidence was 1.8%. The lowest incidence calculated per 100 operations was found for Hospital 1 (H1) and amounted to 1.1%; in Hospital 2 (H2), it was 2.4%; and in Hospital 3 (H3), it was the highest and reached 3.4%. (Table 1).

In total, 2182 HPRO operations were performed. The incidence was 2.1%. The average patient waiting time (from admission until operation) was 5 days. The average length of stay was 13 days and it took 36 days from surgery to the detection of SSI, on average. The mean patient age was 70 years. There were mostly women: 61.2% vs. 38.8% of men. The median HPRO surgery duration was 90 min. Most SSIs for HPRO were detected before discharge from the hospital (52.2%), while after discharge, it was 47.8% (Table 2).

In total, 558 KPRO operations were conducted. The incidence was 2.0%. The average patient waiting time was 5 days. The average duration of hospitalisation was 13 days and it took 52 days from surgery to the detection of SSI, on average. The mean patient age was 69 years. Among patients, 74.4% were women and 25.6% were men. The median KPRO surgery duration was 103 min. Before discharge, seven cases (63.6%) were detected and after discharge, the percentage was 36.4% (*p* = 0.260) (Table 2).

Moreover, 2133 FX operations were carried out. The incidence was 1.9%. The average patient waiting time was 3 days. The average duration of hospitalisation was 9 days and it took 69 days from surgery to the detection of SSI, on average. The mean patient age was 58 years. Among patients, 53.6% were women and 46.4% were men. The median FX surgery duration was 70 min. Additionally, 57.5% received the diagnosis before discharge, and 42.5% after discharge (Table 2).

A total of 1388 CR surgeries were conducted. The incidence was 1.0%. The average patient waiting time was 4 days. The average duration of hospitalisation was 6 days and it took 36 days from surgery to the detection of SSI, on average. The mean patient age was 54 years. Women comprised 46.6% and men 53.2%. The median CR surgery duration was 55 min. Before discharge, 57.1% were diagnosed, and after discharge: 42.1% (Table 2).

The standardized infection ratio (SIR) of HPRO was exceeded (above one) in all studied wards; SIR = 1.9. In order to carry out a detailed analysis of HPRO operations, SIR was calculated for each of the wards studied. In H1, for HPRO surgeries without risk factors, SIR was 1.3, with one risk factor: 1.2, and with two risk factors: 0.8. In H2, for HPRO procedures without risk factors, SIR was 3.2, with one risk factor: 3.5, and with two risk factors: 3.2. In H3, for HPRO operations without risk factors: SIR was 1.2, with one risk factor: 2.9, and with 2 risk factors: 0.0 (Table 3).

SIR was exceeded (above one) for KPRO in all examined wards, with SIR = 3.3 (concerns two wards; in hospital H2, KPRO surgeries were not performed). In H1, SIR for KPRO without risk factors was 1.4, with one risk factor: 5.2, and with two risk factors: 3.9. In H3, for operations without risk factors, SIR was 6.8, and for surgeries with one and two risk factors, it was 0.0 (Table 3).

SIR was exceeded (above one) in FX in two out of three studied wards (H1 = 0.4, H2 = 1.5, and H3 = 7.9). In H1, SIR for FX without risk factors was 0.5, with one risk factor: 0.6, and with two risk factors: 0.0. In H2, for operations without risk factors, SIR was 1.4, with one risk factor: 1.7, and with two risk factors: 1.2. In H3, for operations without risk factors, SIR was as high as 16.8, with one risk factor: 9.6, and with two risk factors: 0.0 (Table 3).

The predominant etiological agents in SSI-HPRO were coagulase-negative *Staphylococcus* and *Klebsiella pneumoniae* comprising 17.4% each. In SSI-KPRO, coagulase-negative *Staphylococcus* (36.4%) was dominant, while Staphylococcus aureus dominated in SSI-FX (42.5%), and in SSI-CR, it was also Staphylococcus aureus (35.7%) (Table 4). All staphylococci were sensitive for vancomycin. In case of E. coli, resistance for ampicillin reached 44.4%. Resistance of *K. pneumoniae* strains for cephalosporins reached 41.7%. Highest resistance was observed in case of A. baumannii—80% for all tested antibiotics; however, only four such strains were isolated. Antibiotic groups most commonly used for treatment of SSI were beta-lactams—73.5%.

## 4. Discussion

HPRO surgery was the most common procedure type and the results of European studies also indicate a high frequency of this type of operation [14,16]. In the study period, 46 cases of SSI HPRO were diagnosed and the incidence of SSI HPRO was 2%. The average incidence of SSI HPRO in European countries was 1.0%, with significant variation in incidence between individual countries, i.e., from 0.4% (Lithuania and northern Ireland) to 2.2% (Norway) [14,16]. Therefore, the incidence obtained in our study (2014–2019) is placed in the upper limit for incidence detected in European countries in 2016–2017. In a single-centre study carried out in Poland by Pawłowska et al. and making use of a seven-year observation in 2009–2016 [5], the incidence of SSI for HPRO was considerably higher and amounted to 6%. However, it should be noted that in our study, the incidence of SSI for HPRO varied in the studied hospitals and the highest incidence reached 4% (H2). It seems that we should investigate reasons for this condition in the quality of supervision of HAI. The examined hospitals took part in the national system of active registration of HAI, according to the ECDC’s HAI-Net methodology, and the hospital with the lowest incidence (H1) also conducted scientific activities with respect to supervision of HAIs, which was the motivation behind the suggestion to improve the system of supervision. Furthermore, in Poland, there are few studies based on the ECDC’s HAI-Net and CDC’s NHSN methodology; hence, it is difficult to conduct discussions involving this topic. However, it seems that participation in organised programmes of surveillance brings measurable benefits in the form of lower HAI rates. The median age of patients with HPRO in Europe (72 years) was comparable with that obtained in our study (71 years) [14,15], which may prove that the health condition of Polish patients as well as their access to HPRO treatment are comparable. Additionally, the percentages of women and men in HPRO procedures were identical to those in the European multi-centre study (M/K 0.6) [14,16]. In our study, comparable numbers of cases of SSI were diagnosed before discharge and after discharge (52% vs. 48%). Investigations in one of the Polish wards studied by Kołpa et al. [17], carried out during two separate time periods, found the percentage of diagnoses before discharge to be 31% (2009–2013), and with an improvement in the pre-discharge detection, in subsequent years, it was up to 50% (2014–2018). The improvement in pre-discharge detection was associated with the introduction of the ECDC’s HAI-Net- into the hospital in 2012. In our study, the median HPRO surgery duration was 90 min, while in European countries, it was 70 min [14,16], which could have had an impact on the risk of developing SSI, since the duration of surgery is one of the main risk factors of developing SSI; it is also included in the SIR. The results of our study according to the SIR-SSI index in HPRO operations are concerning, as in all three hospitals, they exceeded the value of one, which means that it is necessary to take actions that would reduce the incidence of SSI in HPRO procedures. In the examined wards, regarding HPRO surgeries, the most common pathogens were coagulase-negative Staphylococcus 17% and *Klebsiella pneumoniae* 17% as well as Escherichia coli 11%. In the studies conducted by ECDC in 2017, the most frequent pathogen was Staphylococcus aureus (32%) followed by coagulase-negative Staphylococcus (19%) [10]. Differences concerning the detected etiological agents of SSI should be the subject of separate analyses on the local epidemiological situation and possible outbreaks.

Another procedure that was performed in the hospitals studied by us was KPRO surgery, for which the average incidence was 2%. In European studies carried out in 2017, the incidence of SSI KPRO was four times lower, on average, and amounted to 0.5% [15], but there were considerable disparities regarding incidence between individual European countries, from 0.2% (northern Ireland) to 2.7% (Hungary). In the already mentioned single-centre study conducted by Pawłowska et al. [5] in 2009–2016, the incidence of SSI KPRO was even higher, at 5%. In consideration of this state of affairs, a reference should be made to the discussion above with respect to SSI HPRO, as here, lack of participation of the hospital in the HAI-Net methodology programme could similarly have been the reason behind the differences regarding incidence. Nonetheless, it should be emphasised that the mere fact of designing and publishing this study [5] significantly contributes to the assessment of the epidemiological situation in the Polish orthopaedics and trauma wards as other hospitals do not take such actions and perhaps do not know their incidence, and hence do not take preventive measures. In the present study, the median age of KPRO patients was 70 years and it was identical to that in the investigations carried out in Europe in 2017 [14]. In the previous studies conducted in Poland, the median age was 67 years, both in the studies conducted earlier (2008–2012) by Wałaszek et al. [18] and in the later study (2009–2016) by Pawłowska et al. [5]. Hence, it can be cautiously concluded that the health condition of the Polish population is improving with reference to longevity in health. In our study, women were predominant in KPRO procedures and the rate was similar in the European countries (0.7) [14,16]. Our median KPRO surgery duration was 103 min, and in Europe, it was 75 min [14,16]. KPRO operation duration was longer in Polish investigations than in Europe, which could have had an impact on the incidence detected.

In the period under study, there were 64% of SSI cases detected before discharge regarding KPRO surgeries. In the ECDC study [10], the proportion of SSIs diagnosed at the hospital was 12%. Perhaps, also in this case, the longer hospital stays in the Polish wards following KPRO surgeries contributed to the earlier diagnoses of SSIs. Regarding KPRO procedures, the investigated hospitals had SIR rates above one, which means than the risk of acquiring SSI during KPRO operations in the wards studied was higher than in European countries [14]. In our study, the most common etiological agent of SSI for KPRO was coagulase-negative Staphylococcus responsible for 36%, and in the second place, Staphylococcus aureus with 37%. In multi-centre studies conducted in Europe by the ECDC in 2016 and 2017, the dominant pathogen was Staphylococcus aureus [14,16].

In FX operations, the incidence was 2%. The obtained incidence of SSI-FX from our study was compared with American multi-centre studies [18] by calculating SIR. The SIR in H2 and H3 was higher than “1” at all three levels, and in H3, without risk factors, it was over 16 times higher. It means that the risk of acquiring SSI after FX surgeries in some Polish hospitals (H2 and H3) was considerably higher than in the American examination. In H1, this form of infection, SSI-FX, was put under supervision in 2008, which probably contributed to the reduction in the incidence from 3% (in 2008–2012) [18] to the one obtained in this study, 0.4% (2014–2016).

In CR operations, the incidence was 1%. Meanwhile, in the studies conducted by Kołpa et al., the incidence of SSI CR was 2% in 2009–2013, and in subsequent years (2014–2018), it was possible to reduce the ratio to 1% [17]. This effect was achieved through efficient long-term supervision of CR surgeries. CR procedures comprised from 20% to 32% of all operations performed in the wards examined and were subjected to analysis as it was noticed that they may represent a significant proportion of SSIs among orthopaedic operations. However, in the literature, this topic is underresearched and poorly described; thus, it requires further investigation.

To summarise the discussion above, the hospital which conducted continuous long-term targeted surveillance of HAIs, based on the standardized HAI-Net programme, and was involved in scientific activities concerning the supervision of HAIs, had lower rates of HAI. Actions should, therefore, be undertaken to create a network of hospitals to join the infection control programme at regional and national levels. It is crucial, at the national level, to introduce a unified case definition as well as standards of conduct regarding HAI monitoring. Owing to these actions, it can be possible to obtain reliable data from targeted surveillance concerning certain forms of SSI, and by the same token, preventive measures in this respect can be taken.

## 5. Limitations of the Study

The limitation of our study is the small number of settings providing data. The data were not externally validated, which also should be stated as a limitation. A possible limitation is also the approach to analysis itself, although it was based on ECDC’s HAI-Net protocol. This protocol includes a set of collected data, and thus the available outcomes are predetermined. However, it is also an advantage because it enables country-wide or even international benchmarking. There are other factors correlated with orthopaedic surgical procedures, which may affect the incidence rates, such as the type of antibiotic prophylaxis, number of procedures of given and the type performed by the surgeon, and rehabilitation procedures. These are outside of the range of the HAI-Net SSI protocol (version used in this study). Another limitation of this study is also the lack of information of patients’ post medical history as comorbidities.

## 6. Conclusions

HPRO, KROR, and FX operations conducted in the studied wards were associated with a higher risk of developing SSI than that predicted by SIR. Actions should be undertaken to reduce the incidence of SSI following these procedures.

SSI after CR surgeries comprised a significant percentage of the total infection pool.

Results of our study were based on surveillance data from three orthopaedic wards, and thus may not be optimal for assessing the situation at the country level. A unified surveillance system would facilitate infection control and the implementation of procedures to increase patient safety.

## Figures and Tables

**Table 1:** Number, type, and incidence of SSI considering variables: sex, age, and surgery type, in the studied hospitals in 2014–2018.

Data Type	H1	H2	H3	Total
Patients’ age				
Mean age (years)	63.4	59.0	64.2	62.5
Patients’ sex				
Men *n* (%)	1614 (43.3)	658 (43.9)	448 (43.4)	2720 (43.5)
Women *n* (%)	2115 (56.7)	840 (56.1)	585 (56.6)	3540 (56.5)
Total *n* (%)	3729 (100.0)	1498 (100.0)	1033 (100.0)	6260 (100.0)
Type of SSI				
SSI-S *n* (%)	0 (0.0)	5 (13.9)	10 (28.6)	15 (13.5)
SSI-D *n* (%)	40 (100.0)	26 (72.2)	24 (68.6)	90 (81.1)
SSI-O *n* (%)	0 (0.0)	5 (13.9)	1 (2.9)	6 (5.4)
Total SSI *n* (%)	40 (100.0)	36 (100.0)	35 (100.0)	111 (100.0)
Procedure type				
CR *n* (%)	727 (19.5)	330 (22.0)	331 (32.0)	1388 (22.2)
FX *n* (%)	1354 (36.3)	675 (45.1)	103 (10.0)	2132 (34.1)
HPRO *n* (%)	1228 (32.9)	493 (32.9)	461 (44.6)	2182 (34.9)
KPRO *n* (%)	420 (11.3)	0 (0.0)	138 (13.4)	558 (8.9)
Total *n* (%)	3729 (100.0)	1498 (100.0)	1033 (100.0)	6260 (100.0)
Operation time—median				
CR	50.0	35.0	90.0	55.5
FX	70.0	80.0	90.0	75.9
HPRO	85.0	90.0	90.0	88.1
KPRO	105.0	0.0	90.0	99.0
Total	75.0	80.0	90.0	77.6

SSI—surgical site infection, SSI-S—superficial incisional SSI, SSI-S—organ-space SSI, SSI-D—deep SSI, HPRO—hip prosthesis, KPRO—knee prosthesis, FX—open reduction of fracture, and CR—closed reduction of fracture with internal fixation.

**Table 2:** Number and incidence of SSI considering variables: days of waiting for surgery, length of stay in the ward, number of days from surgery to the detection of SSI, patient age, patient sex, ASA score, surgical site cleanliness, duration of surgery, and detection of SSI before or after discharge for HPRO, KPRO, FX, and CR in 2014–2018.

Operation Type	HPRO	KPRO	FX	CR	Total
Number of operations	2182	558	2133	1388	6261
Number of SSI	46	11	40	14	111
Incidence (%)	2.1	2.0	1.9	1.0	1.8
Waiting time for surgery in the ward (days)					
mean; SD; (95% CI)	4.3; 8.5; (3.9–4.7)	4.1; 4.9; (3.6–4.5)	2.6; 5.1; (2.4–2.8)	3.5; 4.7; (3.3–3.8)	3.1; 4.2; (2.9–3.1)
Person–days of hospitalisation in the ward (days)					
mean; SD; (95% CI)	12.5; 10.7; (11.9–13.2)	12.9; 9.5; (11.9–13.8)	9.1; 13.8; (8.3–9.7)	6.1; 6.7; (5.6–6.6)	10.1; 11.8; (9.7–10.5)
Number of days from surgery to the detection of SSI					
mean; SD; (95% CI)	36.1; 69.8; (15.9–56.1)	52.3; 85.3; (8.5–109.6)	68.9; 67.5; (47.3–90.5)	35.5; 20.6; (23.6–47.5)	48.1; 66.7 (45.6–69.4)
Patient age (years)					
mean; SD; (95% CI)	70.9; 11.8 (70.4–71.3)	69.1; 8.2; (68.4–69.7)	57.8; 21.7; (56.9–58.8)	53.6; 26.2; (52.2–54.9)	62.8; 17.7; (62.4–63.1)
Sex					
Sex ratio (M:F)	0.6	0.4	0.7	1.1	0.8
Duration of surgery (minutes)					
50 (percentile)	90	103	70	55	
75 (percentile)	105	120	90	75	
Diagnosis of SSI: before discharge vs. after discharge					
Before discharge *n* (%)	24 (52.2)	7 (63.6)	23 (57.5)	8 (57.1)	63 (54.3)
After discharge *n* (%)	22 (47.8)	4 (36.4)	17 (42.5)	6 (42.9)	53 (45.7)
Total *n* (%)	46 (100.0)	11 (100.0)	40 (100.0)	14 (100.0)	116 (100.0)
Type of SSI					
SSI-S	0 (0.0)	1 (10.0)	10 (25.0)	5 (35,7)	16 (14,4)
SSI-D	46 (100.0)	10 (90.0)	26 (65.0)	9 (64,2)	91 (81,9)
SSI-O	0 (0.0)	0 (0.0)	4 (10.0)	0 (0.0)	4 (3,6)
	46 (100.0)	11 (100.0)	40 (100.0)	14 (100.0)	111 (100.0)

*n*—number, %—percent, HPRO—hip prosthesis, KPRO—knee prosthesis, FX—open reduction of fracture, CR—closed reduction of fracture with internal fixation, ASA—the American Society of Anesthesiologists’ physical status classification, SSI-S—superficial incisional SSI, SSI-S—organ-space SSI, SSI-D—deep SSI, 95% CI—confidence intervals, and SD—standard deviation.

**Table 3:** Incidence of SSI for HPRO and KPRO divided into three orthopaedic hospitals in comparison to ECDC 2017 [14] and FX-NHSN [15] considering risk factors in 2014–2018.

Hospital Type	Number of Surgeries	Number of SSIs	Incidence (%)	Incidence (%) ECDC	Expected Number of SSIs	SIR * SSI	SD; (95%CI)
			HPRO—Hip Prosthesis				
Hospital (H1)	1228	16	1.3	1.1	15	1.1	1.2; (0.2–5.2)
Hospital (H2)	493	18	3.7	1.1	5	3.5	
Hospital (H3)	2182	46	2.1	1.1	24	1.9	
KPRO—Knee Prosthesis							
Hospital (H1)	420	9	2.1	0.6	3	3.0	0.4; (0.2–6.5)
Hospital (H3)	138	2	1.4	0.6	1	2.4	
FX—Open Reduction of Fracture							
Hospital (H1)	1354	9	0.7	2.1	21	0.4	4.1; (0.3–13.3)
Hospital (H2)	675	14	2.1	2.1	9	1.5	
Hospital (H3)	103	17	16.5	2.1	2	7.9	

*SIR—standardized infection ratio.

**Table 4:** Etiological agents of SSI-D in 2014–2018 in orthopaedic wards.

	HPRO	KPRO	FX	CR	Total *n* (%)
No etiological agent *n* (%)	1 (2.2)	2 (18.2)	3 (7.5)	0 (0.0)	6 (5.4)
Not detected/not collected	1 (2.2)	2 (18.2)	3 (7.5)	0 (0.0)	6 (5.4)
Gram-positive cocci *n* (%)	16 (34.8)	7 (63.6)	24 (60.0)	7 (49.9)	54 (48.6)
*Staphylococcus aureus*	4 (8.7)	3 (27.3)	17 (42.5)	5 (35.7)	29 (26.1)
*Coagulase-negative staphylococci*	8 (17.4)	4 (36.4)	3 (7.5)	1 (7.1)	16 (14.4)
*Enterococcus faecalis*	4 (8.7)	0 (0.0)	2 (5.0)	0 (0.0)	6 (5.4)
Other *Gram-positive cocci*	0 (0.0)	0 (0.0)	2 (5.0)	1 (7.1)	3 (2.7)
Enterobacteriaceae *n* (%)	18 (39.1)	1 (9.1)	7 (17.5)	4 (28.5)	30 (27.0)
*Escherichia coli*	5 (10.9)	0 (0.0)	2 (5.0)	2 (14.3)	9 (8.1)
*Klebsiella pneumoniae*	8 (17.4)	1 (9.1)	3 (7.5)	0 (0.0)	12 (10.8)
Other *Enterobacteriaceae*	5 (10.9)	0 (0.0)	2 (5.0)	2 (14.3)	9 (8.1)
Non-fermenting Gram-negative bacteria	11 (23.9)	1 (9.1)	4 (10.0)	3 (21.4)	19 (17.1)
*Acinetobacter baumannii*	3 (6.5)	1 (9.1)	0 (0.0)	1 (7.1)	5 (4.5)
*Proteus mirabilis*	5 (10.9)	0 (0.0)	4 (10.0)	2 (14.3)	11 (9.9)
Other non-fermenting *Gram-negative bacteria*	3 (6.5)	0 (0.0)	0 (0.0)	0 (0.0)	3 (2.7)
Others	0 (0.0)	0 (0.0)	2 (5.0)	0 (0.0)	2 (1.8)
*Clostridium* spp.	0 (0.0)	0 (0.0)	1 (2.5)	0 (0.0)	1 (0.9)
*Corynebacterium* spp.	0 (0.0)	0 (0.0)	1 (2.5)	0 (0.0)	1 (0.9)
Total *n* (%)	46 (100.0)	11 (100.0)	40 (100.0)	14 (100.0)	111 (100.0)

## Data Availability

Data are available upon reasonable request from Marta Wałaszek.
